# Induction of central nervous system plasticity by repetitive transcranial magnetic stimulation to promote sensorimotor recovery in incomplete spinal cord injury

**DOI:** 10.3389/fnint.2014.00042

**Published:** 2014-05-20

**Authors:** Peter H. Ellaway, Natalia Vásquez, Michael Craggs

**Affiliations:** ^1^The London Spinal Cord Injury Centre, Royal National Orthopaedic HospitalStanmore, UK; ^2^Division of Brain Sciences, Centre for Clinical Neuroscience, Imperial College LondonLondon, UK; ^3^Division of Surgery and Interventional Sciences, University College LondonLondon, UK

**Keywords:** spinal cord injury, repetitive transcranial magnetic stimulation, corticospinal, neural plasticity, sphincter muscle, pudendal anal reflex

## Abstract

Cortical and spinal cord plasticity may be induced with non-invasive transcranial magnetic stimulation to encourage long term potentiation or depression of neuronal circuits. Such plasticity inducing stimulation provides an attractive approach to promote changes in sensorimotor circuits that have been degraded by spinal cord injury (SCI). If residual corticospinal circuits can be conditioned appropriately there should be the possibility that the changes are accompanied by functional recovery. This article reviews the attempts that have been made to restore sensorimotor function and to obtain functional benefits from the application of repetitive transcranial magnetic stimulation (rTMS) of the cortex following incomplete spinal cord injury. The confounding issues that arise with the application of rTMS, specifically in SCI, are enumerated. Finally, consideration is given to the potential for rTMS to be used in the restoration of bladder and bowel sphincter function and consequent functional recovery of the guarding reflex.

## INTRODUCTION

An injury to the spinal cord may create sensory and motor loss or impairment that is likely to be permanent and can be severe enough to significantly impair quality of life. Natural recovery is limited (Fawcett et al., 2007) and treatments to aid recovery have in the main provided rather modest functional benefits. Approaches to restoration of function have focused on surgery, drug administration, cell-based treatments, recovery of axonal transmission and rehabilitation or combinations of these approaches. This article will focus on rehabilitation directed at cortical and spinal cord plasticity which may be induced with non-invasive electrical and magnetic stimulation techniques that create long term potentiation or depression of neuronal circuits (Oudega and Perez, 2012). Such plasticity inducing stimuli provide attractive approaches to promote beneficial changes in motor circuits that have been degraded by spinal cord injury (SCI). If residual circuits can be conditioned appropriately there could be a possibility of the changes being accompanied by functional recovery. However, this statement should be qualified by the following considerations.

The focus here will be on the corticospinal pathway, but it should be remembered that voluntary motor acts frequently require corticospinal drive to be accompanied by activity in sub-cortical structures (vestibulospinal, reticulospinal) for stabilization and balance. These pathways are likely to be compromised along with the corticospinal tract in SCI. Reparative methods specifically targeting corticospinal circuitry might produce changes that do not result in integrated functional recovery. Ultimately, methods to augment circuits linked to these sub-cortical structures (Chen et al., 2003; Zaaimi et al., 2012; Weishaupt et al., 2013) may need to be combined with those addressing the corticospinal system. A further caveat is that, while conditioning with repetitive electrical or magnetic stimulation may result in altered central neural activity, even to the extent of restoring conduction in motor pathways, it may not be sufficiently specific to effect beneficial functional outcomes that are normally based on coordinated timing in complex circuitry. The recent success using epidural electrical stimulation to restore some voluntary movement in human complete SCI does however, reveal the potential for such methods to reveal latent corticospinal pathways in clinically confirmed (AIS A and B), complete motor paralysis (Angeli et al., 2014). Additionally, it may be argued that expectations of functional recovery would be higher if any “treatment” were combined with task specific rehabilitation. Indeed, this may be why combination therapies such as digestion of chondroitin sulfate proteoglycans coupled with voluntary tasks, which are thought to be synergistic and non-interfering, has been effective (García-Alías and Fawcett, 2012). However, other combination approaches that were expected to be synergistic have failed to produce levels of recovery greater than those gained by independent application of the individual treatments, e.g., the anti-Nogo antibody combination with treadmill training in rats (Maier et al., 2009). It seems likely that the inherent plasticity of the nervous system may even produce competing neural changes that are maladaptive with combinatorial approaches. Clearly, such approaches in patients will need to proceed with caution. There appears to have been only one study combining an repetitive transcranial magnetic stimulation (rTMS) protocol with task-specific training. Following stroke, rTMS induced a transient increase in cortical excitability of the lesioned hemisphere but did not prove to be an adjunct to task-specific training of the arm (Higgins et al., 2013).

In this contribution to mechanisms of motor function recovery after SCI the potential of rTMS to facilitate motor recovery after incomplete spinal injury (iSCI) is explored by reviewing the studies conducted so far and by considering the limitations to be expected that may be peculiar to the specific condition of SCI. Most attempts to facilitate recovery of sensorimotor control in human iSCI and in animal models of SCI have focused on limb musculature. This fits with the principal priority for recovery for those with tetraplegia which is restoration of hand and arm function (Anderson, 2004; Snoek et al., 2004). However, improvements in bladder and bowel function emerge as clear priorities over any other impairment with regard to enhancement of quality of life for paraplegics, and rank second only to restoration of hand function even for tetraplegics. Attempts to restore bladder and bowel function in iSCI (Grill et al., 2001; Craggs, 2006; de Groat and Yoshimura, 2006) have received less attention than for either upper or lower limb function. Rather, the approach in SCI has been long-term and continuous management of continence and voiding using pharmacological, catheterization, or electrical stimulation techniques. Restoration of sphincter function and the promotion of continence might however be amenable to neural plasticity inducing approaches through activity dependent rehabilitation (Lynskey et al., 2008). Any novel strategy for achieving this aim would benefit from knowing the extent to which individuals with iSCI have retained voluntary control of sphincter musculature. Equally, due to the considerable variation in impact of SCI on pelvic floor control, electrophysiological estimates of residual function of the corticospinal tract innervating pelvic musculature and of the status of pudendal reflexes (hyporeflexia, hyper-reflexia, dyssynergia) may be required to tailor specific protocols. The final section of this article describes one attempt to establish such criteria as a preliminary to developing new treatment for restoration of sphincter function.

## rTMS AND CORTICAL EXCITABILITY

The basis for recovery of motor function from neurological trauma or disease by the use of repetitive non-invasive transcranial magnetic stimulation (TMS) is likely to be the induction of cortical and/or spinal cord plasticity. Such stimulation is known to produce long term potentiation or depression of neuronal circuits depending on the exact protocol of stimulation employed. This means that selection of the parameters for stimulation can, potentially, provide functional benefits by the appropriate raising or lowering of excitability of circuits determining motor behavior. Such plasticity inducing stimuli provide attractive approaches to promote beneficial changes in motor circuits that have been degraded by SCI. If residual circuits can be conditioned appropriately there should be the possibility that the changes are accompanied by functional recovery.

There is a substantial literature documenting the effects of rTMS on human motor cortical excitability (Fitzgerald et al., 2006). Low frequency rTMS of the order of 1Hz or less tends to effect a reduction in cortical excitability whereas higher frequency rTMS (≥5 Hz) is mostly reported as causing increased excitability of the corticospinal pathway and a reduction in cortical inhibition. However, there is inconsistency among reports and it is evident that any effect of rTMS on motor cortical excitability depends on factors in addition to frequency, such as total number of pulses, pattern, duration and strength of stimulation (Fitzgerald et al., 2006) and by attention to the process (Stefan et al., 2004). A further complication, albeit one that may be manipulated to advantage when the intention is to promote recovery of function, is that rTMS effects are heavily modified by prior or parallel voluntary motor activity or by intentional priming using alternative non-invasive stimulation (Ridding and Ziemann, 2010).

Combining repetitive motor cortical stimulation with peripheral nerve stimulation may also induce plasticity in motor circuits if the interval between the two types of stimulation is appropriate. This has its basis in the Hebbian theory of spike-timing-dependent plasticity and has been applied to the human central nervous system by the use of us of paired associative stimulation (PAS; Stefan et al., 2000). The concept is that if two inputs are repeatedly paired to arrive at a neuronal circuit within a short space of time of each other then the connectivity of synapses involved will alter and the change be sustained beyond the period of treatment. The direction of change (facilitation or inhibition) depends on the timing. In general, if one input arrives shortly before, or synchronous with, another then the action of the latter will be potentiated (Wolters et al., 2003; Ridding and Rothwell, 2007). A later arrival time is likely to lead to depression of neurons in the circuit. The first application of the PAS protocol involving cortical stimulation employed a peripheral nerve stimulus delivered in advance of a cortical TMS pulse with the paired stimuli delivered at low rates (0.05 Hz) for a period of minutes or more (Stefan et al., 2000). The interval between peripheral nerve stimulus and cortical stimulus was selected such that the afferent nerve volley elicited by the peripheral stimulus arrived at the sensorimotor cortex approximately synchronously with the TMS pulse. The protocol induced an increase in excitability of the motor cortex evident as an increase in amplitude of motor evoked potentials (MEPs) in muscles whose motor cortical representation had been targeted by the cortical stimulation during the PAS treatment. Alternatively, the PAS strategy can target synapses in the spinal cord at the level of the motoneurons. Taylor and Martin (2009) showed that lasting alteration of cortico-motoneuronal synapses in the spinal cord may be achieved by judicial timing of paired cortical and peripheral (antidromic motoneuronal) nerve stimuli. Conditioning intervals that increased or decreased MEPs elicited by cervico-medullary delivery of test TMS similarly increased or decreased voluntary force and electromyography (EMG) for the peripheral muscle (biceps brachii) tested.

PAS and rTMS applied to the human brain have lasting effects but most paradigms to date have produced changes that persist for the order of minutes rather than days or longer. The prospect for longer lasting effects that might translate to durable functional gains waits further development of the techniques and studies on the safety of long-lasting brain stimulation.

## rTMS AND PAS IN SPINAL CORD INJURY

Repetitive forms of TMS have been extensively used as putative remedial treatments in clinical neurology particularly for psychiatric conditions (Aleman, 2013) and for pain (Nardone et al., 2013), but also with limited success in disorders affecting motor control including stroke (Hao et al., 2013), Parkinson’s disease (Udupa and Chen, 2013), and dystonia (Kimberley et al., 2013). Their use in SCI has been less extensive. Most applications have been with the intention of relieving neuropathic pain (Nardone et al., 2013) but the following applications have addressed motor control.

### SENSORIMOTOR CONTROL

To date, five studies have reported on the effects of applying repetitive TMS with intent to modulate sensorimotor control in iSCI. Four studies have employed high frequency (>5 Hz) rTMS, and one study has used a PAS protocol (**Table 1**).

**Table 1:** Review of the effects of rTMS and PAS on sensorimotor function and spasticity in spinal cord injury.

Study	Number in study	AIS and level	Trial protocol	TMS intensity	Freq. of TMS (Hz)	Total pulses	Target	Outcome timing	Outcomes
***Sensorimotor function***
Belci et al. (2004) rTMS	4	AIS D, C5	Placebo, random., X-over SB	90% RMT	10 + 0.1 Hz, doublets	360 doublets × 5 (days)	UL	Week of treatment	↑PP, ↑U&LEMS, ↓EPT, ↓Peg board time, ↓Cortical Inhibition
								Follow-up 3 weeks	↑PP, ↑U&LEMS, ↓EPT, ↓Peg board time persisted
Kuppuswamy et al. (2011), rTMS	15	AIS A–D, C2–C8	Placebo, random., X-over SB	80% AMT	5 Hz	900** ×** 5 (days)	UL	1, 72, and 120 h post rTMS	ASIA no change, ↑ARAT at 1h, ↑AMT at 72 and120 h, ↓EPT persisted (2 weeks) in two subjects
Benito et al. (2012), rTMS	17	AIS D, C4–T12	Placebo, random, X-over. DB	90% RMT	20 Hz	1600 × 15 (days)	LL	Post rTMS	↑ LEMS = WISCI-II, ↑10MWT, ↑cadence (↑step length and ↓TUG no difference to sham)
								Follow-up 2 weeks	↑10MWT sustained over sham
Kumru et al. (2013)****^†^, rTMS	10	AIS D, C4–T12	Placebo, random, X-over. SB	90% RMT, (UL muscle)	20 Hz	1600 × 15 (days)	LL	Post rTMS	↑LEMS, ↑10MWT** =** WISCI-II = TUG
								Follow-up 2 weeks	↑10MWT sustained over sham
Bunday and Perez (2012) ^††^, PAS	19	AIS A–D, C4–C8	X-over. SB	100% MSO	0.1 Hz	~100	UL	0–30 min post PAS	↑MEP, ↑cMEP, ↑voluntary force, ↓9HPT = F-waves
								1–2 h	↑MEP
***Spasticity***
Kumru et al. (2010), rTMS	14	AIS C–D, C4–T12	Placebo, random., X-over. DB	90%RMT, (Biceps brachii)	20 Hz	1600 × 5 (days)	LL	During and post rTMS sessions	Less spasticity, ↓MAS, SCAT & MPSFS = Hmax/Mmax, = T reflex = withdrawal reflex
								Follow-up 1 week	Reduction in spasticity persisted
Benito et al. (2012), rTMS	17	AIS D, C4–C12	Placebo, random., X-over. DB	90% RMT	20 Hz	1600 × 15 (days)	LL	Post rTMS	Less spasticity, ↓MAS
Kumru et al. (2013)^†^**,** rTMS	10	AIS D, C4–T12	Placebo, random., X-over. SB	90%RMT, (UL muscle)	20 Hz	1600 × 15 (days)	LL	Post rTMS	Less spasticity (↓MAS)

*AIS, American Spinal Injuries Association (ASIA) Impairment Scale; AMT, active motor threshold to TMS; DB, double blinded; Hmax/Mmax, ratio of maximum H reflex to maximum M wave; LL, lower limb; MAS, Modified Ashworth Scale; MPSFS, Modified Penn Spasm Frequency Scale; MSO, maximal stimulator output; PAS, paired associative stimulation; PP, ASIA pin prick score; Random, randomised; RMT, resting motor threshold to TMS; rTMS, repetitive transcranial magnetic stimulation; SB, single blinded; SCAT, Spinal Cord Assessment Tool for Spasticity; T reflex, tendon reflex; TUG, timed up and go test; UL, upper limb; U&LEMS, upper and lower extremity motor scores; WISCI-II, Walking Index for SCI Scale; X-over, cross-over trial; 9HPT, nine-hole peg-board test. 10MWT, 10 min walking test; =, no change; ↑, increase; ↓, decrease; ^†^Study combined rTMS with gait rehabilitation therapy; ^††^ PAS study consisted of paired cortical TMS (100% MSO) and supramaximal peripheral nerve stimulation.*

In a small population sham-controlled study of four stable iSCI subjects, Belci et al. (2004) used double pulses of TMS separated by 100 ms (10 Hz) at a frequency of 0.1 Hz (10 s interval) for 1 h on each of five consecutive days – effectively, a combination of high and low frequency TMS. They found improvements in clinical measures of motor and sensory function. International standards for neurological classification of spinal cord injury (ISNCSCI) assessments of light touch (LT), pin prick (PP), and combined upper and lower extremity motor scores (U&LEMS) were all elevated during rTMS treatment and at follow-up (3 weeks). The electrical perceptual threshold (EPT) of a dermatome (C6) affected by the injury (C5) was lowered and time taken to complete a peg-board motor task was reduced with significant changes evident into the follow-up period. Additionally, cortical inhibition evident as the silent period in voluntary EMG following single pulse TMS was reduced during the treatment week but was not long lasting. The small numbers involved in this study indicate caution in interpretation and the rTMS protocol does not shed light on which frequency of stimulation or combination of stimuli might have been responsible for the observed results.

As in the Belci study, Kuppuswamy et al. (2011) also targeted the hand and arm representation of the sensorimotor cortex with rTMS (again sham controlled) to see whether clinical, functional and neurophysiological improvements could be achieved for the upper limb in iSCI. The so-termed high-frequency (5 Hz) rTMS regime, proven to facilitate corticospinal drive in uninjured subjects (see Fitzgerald et al., 2006) was employed. The treatment produced no change in clinical ISNCSCI outcome measures. Time to complete a peg-board test was not changed but functional improvement was observed at 1 h post-treatment, as assessed by the action research arm test (ARAT), particularly in the pinch grip. This was not accompanied by changes in corticospinal thresholds for eliciting MEPs to single pulse TMS. Paradoxically, active motor threshold for eliciting an MEP in a hand muscle was increased rather than decreased at 72 and 120 h after rTMS at which time the change in the functional arm test (ARAT) was no longer evident. Although there was no overall change in cutaneous sensory threshold, 2 of 15 individuals showed persistent reductions in EPT. This may be of relevance in assessing the outcome of future interventions. Clearly, the impact of any SCI is peculiar to the individual concerned, with differences in the level and severity of sensory, motor, and autonomic dysfunction presenting particular profiles. It would not therefore be surprising if treatments, such as rTMS, might affect individuals differently. There were no changes to sham rTMS treatment.

In a further study Benito et al. (2012) investigated whether high-frequency (20 Hz) rTMS could improve gait in stable iSCI subjects. The study reported a significant improvement in clinical lower extremity motor scores. Equivocally, gait parameters including the 10 m walk test, cadence, step length, and the timed up and go test (TUG) were improved, with improvements being maintained for 2 weeks, but step length and TUG also improved following sham stimulation. Significantly, the active rTMS failed to produce any change in the walking index for SCI (WISCI II) scale (Ditunno et al., 2007). In an animal study of relevance, 10 Hz rTMS significantly improved the recovery of gait in rats when applied immediately after low thoracic (T10-T11) spinal cord compression injury but not following higher thoracic (T4–T5) injury (Poirrier et al., 2004). This animal study again highlights the difficulty of carrying out controlled studies in SCI where individual variability in the location and extent of lesions may obscure the effect of treatments. In their study Benito et al. (2012) had only one subject with a low thoracic injury (T11), the others being mid thoracic or cervical. This group have now extended their investigation into whether 20 Hz rTMS can improve gait in iSCI (Kumru et al., 2013) by combining periods of active rTMS with gait training rehabilitation sessions (see **Table 1**). Significant improvements were observed immediately after the rTMS sessions for clinical motor assessment (LEMS) and walking speed (10 m walking test) but, again, no improvement was observed on the WISCI II test. Improvement in walking speed was maintained during a 2 week follow-up period. The conclusion from these two studies is that combining the rTMS with active gait rehabilitation did not influence outcome of the 20 Hz rTMS treatment.

Bunday and Perez (2012) employed a PAS protocol to see whether the arrival of a corticospinal volley immediately prior to motoneuron discharge would enhance voluntary motor control in iSCI. Motoneuron discharge was elicited by stimulation of a peripheral nerve eliciting antidromic invasion of action potentials in motoneurons of a hand muscle (flexor digitorum longus). A short period of paired pulse stimulation (100 pairs at 0.1 Hz), timed such that a corticospinal volley arrived 1-2 ms before antidromic invasion of motoneurons, resulted in increased size of test MEPs irrespective of whether the cortical stimulation was magnetic or electrical. The facilitation remained evident 30 min after PAS treatment and returned to baseline after 1-2 h. MEPs elicited by stimulation of the corticospinal tract at the level of the cervico-medullary junction were also enhanced indicating that the persistent facilitation occurred at the level of the spinal cord. However, the amplitude and persistence of F waves remained unchanged suggesting that the facilitation of MEPs was not related to increases in the excitability of spinal motoneurons but at a presynaptic site. Another experiment timing the pairing such that the corticospinal volley arrived 15 ms after antidromic invasion, MEPs were decreased in size. The findings indicate that the process engaged by the PAS was spike timing-dependent plasticity of residual corticospinal-motoneuronal synapses. The findings were replicated in control neurologically normal subjects. Post PAS, completion of a nine-hole per-board task and a measure of voluntary force were both enhanced, suggesting that the spike-timing dependent approach to modulating plasticity of residual cortico-motoneuronal synapses might be developed further to promote functional recovery from iSCI.

### SPASTICITY

Some 65–78% of individuals with chronic, stable SCI are reported to have symptoms of spasticity (Adams and Hicks, 2005) and spasticity may be elicited by movement provocation in 60% of those reporting symptoms (Sköld et al., 1999). Treatment of spasticity in SCI consists mainly of management of the symptoms by continuous pharmacological application or by physiotherapy or electrical stimulation techniques. The duration of the effects of most physical therapies is relatively short (Gracies, 2001) and there appear to be few clear, long-term effects that persist beyond the period of treatment (Aydin et al., 2005). Patients suffering from spasticity have exaggerated H-reflexes (Morita et al., 2001; Crone et al., 2003). Since application of 5 Hz rTMS has been found to increase pre-synaptic inhibition and thereby decrease H-reflexes (Perez et al., 2005) it is relevant to consider whether rTMS might alleviate the condition of spasticity. Some success has already been obtained in patients diagnosed with multiple sclerosis. A 5 Hz rTMS protocol decreased the H-reflex to M-wave ratio for the soleus muscle and, when repeated during a 2 week period, rTMS produced long-lasting (at least 1 week) clinical improvement in spasticity of lower limbs (Centonze et al., 2007).

In a study of the efficacy of rTMS in reducing spasticity in iSCI (Kumru et al., 2010), 5 daily sessions of 20 Hz rTMS applied to the cortical leg motor area produced significant clinical improvement in lower limb spasticity as measured by the Modified Ashworth Scale (MAS) and the spinal cord injury spasticity evaluation tool. Sham rTMS had no effect. The improvement with rTMS lasted for at least 1 week following the intervention but was not accompanied by changes in corticospinal or segmental reflex excitability. In a further study by the same group (Benito et al., 2012) an increased number of daily rTMS sessions (from 5 to 15) of 1600 stimuli at 20 Hz again reduced spasticity as measured by the MAS at the end of the last rTMS session. In a third study by the group (Kumru et al., 2013) frequency of rTMS stimulation, number of stimuli and daily sessions were unchanged from the previous study (Benito et al., 2012) but the rTMS sessions were combined with active gait rehabilitation. Reassuringly, the addition of concurrent rehabilitation exercises did not affect the outcome of reduced spasticity at the end of the last rTMS session. Limited functional benefit was also achieved, as evident from increased 10 m walk times, although the outcome of the WISCI-II test was unchanged. Neither of these last two studies followed up the effect of treatment on spasticity at a later time.

The number of studies that have investigated the effect of rTMS on sensorimotor control in iSCI is low. For that reason, no clearly defined protocols have emerged that produce persistent functional improvements. Neither is it evident that any short term clinical or functional changes are clearly accompanied by appropriate or consistent neurophysiological changes. In summary, there are many factors to be taken into account in such a complex of disorder as SCI before remedial treatments can be identified with any certain expectation that their application may lead to functional recovery. These are considered in the following sections.

## CONFOUNDING ISSUES TO THE USE OF rTMS IN SPINAL CORD INJURY

### HIGH INTENSITIES OF TMS

One issue that is not immediately evident from the more extensive literature on the use of single pulse TMS, as a means of testing the patency of corticospinal pathways in SCI, is the relatively high strength of TMS required to elicit a MEP in muscles innervated below the level of an incomplete lesion. Kuppuswamy et al. (2011) found the active motor threshold required to elicit a MEP in a hand muscle affected by cervical injury to be greater than 70% of maximum stimulator output (% MSO) for five of seven subjects and Freund et al. (2011) found similarly for three of nine subjects. In a study of the lower limb (Kumru et al., 2010) MEPs could be elicited in the tibialis anterior muscle innervated below an incomplete SCI in only 3 of 15 subjects, and only at high strengths of stimulation (60, 90, and 98% MSO). For comparison, in a study of hand muscles in a large population (*n* = 151) of neurologically normal subjects, none of whom were on medication with known CNS effects, Wassermann (2002) found the active motor threshold of a hand muscle to be 38% MSO, and only 2/151 had active thresholds >70% MSO. It has been widely observed for some time that weak voluntary activation of a muscle facilitates the response to motor cortical TMS and that accompanying active motor thresholds are lower than resting motor thresholds (Rothwell et al., 1991); the average resting threshold in the Wassermann (2002) study was 49% MSO. The resting motor cortical threshold for muscles below the level of incomplete injury in many individuals is thus likely to be higher than 70% MSO and may actually be out of range of magnetic stimulators, despite evidence of weak voluntary activity (Kuppuswamy et al., 2011). This may limit the use of rTMS as a remedial treatment on two counts. First, the majority of reports that have deployed high frequency rTMS to elicit sustained increases in motor cortical excitability have used strengths of stimulation at or above 90% of resting or active motor threshold (Fitzgerald et al., 2006; Hoogendam et al., 2010). Such a level of rTMS for muscles below an incomplete SCI is likely to cause discomfort, pain and distress to the subject, as a result of marked facial cutaneous sensation caused when TMS stimulates scalp muscles. Fortunately, the sensations and discomfort of rTMS at high strengths of stimulation are transient and there are no known lasting effects in terms of safety or well-being of subjects (Rossi et al., 2009). Nevertheless, such intense rTMS is likely to be unacceptable for most subjects. Second, the need to deliver high frequencies at high intensities during rTMS rapidly heats magnetic coils and drastically limits the duration over which stimulation can be applied, often down to far less than control studies would indicate to be effective. This may be the reason why some studies (Benito et al., 2012; Kumru et al., 2013) have deployed intermittent bouts of high frequency rTMS that would allow cooling of the coil periodically during treatment. If this intermittent application of rTMS is employed, rather than a continuous period of stimulation, it may be actually be more likely to have an excitatory effect on the motor cortex (Rothkegel et al., 2010) and mitigate the issue of using high intensity TMS.

The high intensities of TMS that may be required for effective corticospinal stimulation of motor circuits below the level of injury in iSCI raise the possibility of inadvertent excitation of deep brain structures that might have undesirable or negative outcomes. There is no compelling evidence for this as yet but Zangen et al. (2005) have stressed caution and the need for future studies to address safety and efficacy of high intensity magnetic stimulators capable of stimulating brain structures deep to the motor cortex.

Despite these apparent contra-indications to the use of rTMS in SCI, low intensities may prove effective in driving neural plasticity at the level of the cortex. The high thresholds required to elicit MEPs in many SCI subjects most likely reflects impaired axonal transmission in the spinal cord, not the level of excitability in the motor cortex. Although adaptive changes may have occurred in the cortical circuitry as a result of SCI there is no reason to expect those circuits to require the high intensities of TMS that would be needed to elicit MEPs.

### SAFETY ISSUES

In general, the safety considerations in applying TMS to the human brain have been considered by Wassermann (1998) and, more recently, by Rossi et al. (2009). There are safety issues that are of particular concern when applying rTMS to subjects with iSCI. Allodynia is a state that may be experienced by those with iSCI (Bryce et al., 2007) so that TMS could evoke contractions that, although innocuous to a non-injured subject, might provoke pain. Further, if rTMS evoked marked contractions in muscles below the level of injury then triggering episodes of autonomic dysreflexia might be a consequence in susceptible individuals. Equally, such contractions would have the propensity to trigger spasms in unrelated muscles in those iSCI subjects prone to spasticity (Biering-Sørensen et al., 2006). Among contra-indications to the use of TMS, in general, in SCI are implanted (cranial) ferromagnetic hardware such as skull plates or cochlear implants where movements of the implant could occur as magnetic fields are induced.

It might be thought that these issues are unlikely to be of real concern since the strength of rTMS typically applied in attempts to induce central nervous system plasticity are applied at just below threshold for producing contraction in the target muscle. However, there is reduced seizure threshold associated with closed head injury (Cohen et al., 2007) and such injury frequently accompanies SCI resulting in tetraplegia (Macciocchi et al., 2008). Reduced seizure thresholds are also produced by K^+^ channel blockers such as 4-aminopyridine commonly administered in SCI. Further, as discussed above, high strengths might be predicated for effectiveness in targeting plasticity of circuits controlling musculature affected by SCI where resting and active motor thresholds are raised well above control (un-injured) states. These target muscles might themselves not produce strong contractions but due to lack of cortical specificity with TMS, other muscles might respond more vigorously.

### INDIVIDUALITY OF RESPONSE TO TREATMENT

A further confounding issue in developing the use of rTMS to promote recovery of function is that some individuals with iSCI may respond whereas others do not. Genetic factors are likely to be a particularly important source of variation in response to rTMS. A polymorphism in the gene encoding brain-derived neurotrophic factor prevents modification of use-dependent plasticity in the motor cortex in roughly one third of subjects (Kleim et al., 2006) and it is quite possible that other genetic abnormalities may influence the capacity for cortical plasticity in individuals (Ridding and Rothwell, 2007). A recent study Missitzi et al. (2011) found inter-pair differences in adaptive changes in MEPs were about double for dizygotic twins compared to monozygotic twins who had received paired associative (TMS/peripheral nerve) stimulation.

Specifically, in a study of the natural neurological progress of recovery of cervical SCI patients with sensory sparing but complete motor paralysis below the level of injury (AIS B), the preservation of pinprick sensation rather than any other characteristic was the best prognostic indicator for useful motor recovery (Katoh and El Masry, 1995). In the application of rTMS to the hand area of the motor cortex reviewed above (Kuppuswamy et al., 2011), transient functional improvement, as assessed by the ARAT, and changes in TMS motor thresholds for MEPs were found across a group of iSCI subjects with cervical (C2–C8) injuries. However, persistent improvements in sensory EPTs were limited to 2 out of 15 individuals. The two subjects showed systematic reductions in EPT following rTMS that persisted through a 2 week wash-out period and were unchanged by subsequent sham stimulation. Finally, a multi-center trial of a large number (>1,000) of SCI patients Whiteneck et al. (2012), in which clinicians delivered standard rehabilitation care, it was found that individual patient characteristics were strong predictors of outcome.

Thus it appears that the impact of any individual spinal cord lesion, even when assessed clinically as having the same AIS grade and level of lesion, is likely to be unique in terms both of its effect and outcome to treatment. It has been noted that such variability poses problems for the recruitment of a homogeneous population for a clinical trial (Tuszynski et al., 2007).

### ADDITIONAL FACTORS

There are several additional factors, unrelated specifically to SCI, that have the potential to affect the outcome of rTMS treatments and these have been reviewed recently (Ridding and Rothwell, 2007). They include genetic disposition, hormonal, and pharmacological factors, diurnal rhythms and even attention, or lack of attention, to the muscles targeted by the treatment. Additionally, Ferguson et al. (2012) review situations where activity dependent plasticity in the spinal cord may be maladaptive and may reduce the future ability of the cord to adapt appropriately for functional recovery or may promote undesirable states such as neuropathic pain.

Any of these factors may have to be considered in the design of clinical trials (Lammertse et al., 2007) by employing rigorous inclusion and exclusion criteria (Tuszynski et al., 2007) in the selection of cohorts of iSCI subjects that participate in further research into possible remedial benefits of rTMS.

The promising, if inconsistent, motor actions of rTMS reviewed above warrant further refinements of both stimulation protocols and inclusion criteria for subjects that might benefit from treatment. Additionally, it is timely to consider whether the neural control of motor performance impaired by SCI other than in the limbs might respond to rTMS. Below we describe preliminary investigations into the interaction between corticospinal drive and reflex control of pelvic sphincter musculature (Craggs et al., 2007; Vasquez et al., 2014).

## TOWARD RESTORATION OF SPHINCTER FUNCTION IN iSCI

Control of the bowel, bladder, sphincters, and pelvic floor relies on the integrity of spinal pathways originating in both the brain stem pontine areas and the cerebral cortex. Following supra-sacral SCI, coordination fails and neurogenic detrusor over-activity (NDO) and detrusor sphincter dyssynergia (DSD) emerge. The bladder guarding reflex is a response which normally helps to maintain continence by increasing tone in the striated urethral sphincter as the bladder fills. It is aberrant in iSCI, with detrusor-sphincter dyssynergia, but the expression of these disordered reflexes (NDO and DSD) cannot be predicted exactly by the nature and neurological level of SCI. The guarding reflex normally prevents urinary and fecal incontinence through involvement of pontine centers and the integrity of supra-sacral spinal pathways and can be facilitated by voluntary control. However, the guarding reflex is absent in over 85% of patients with complete (AIS A) supra-sacral SCI (Siroky and Krane, 1982). Furthermore, at bladder end fill volume, defined as the volume at which NDO occurs, the guarding reflex as measured by the optimized pudendo-anal reflex (PAR; Podnar and Vodusek, 2001), is found to be absent or weak in patients with a neurologically defined complete supra-sacral SCI (AIS A; Craggs, 2006). However, in incomplete lesions (iSCI - AIS B–D) the guarding reflex is often preserved but very variable. Incomplete SCI patients also demonstrate an enhanced PAR during detrusor-sphincter dyssynergia, in contrast to healthy volunteers.

**FIGURE 1:**
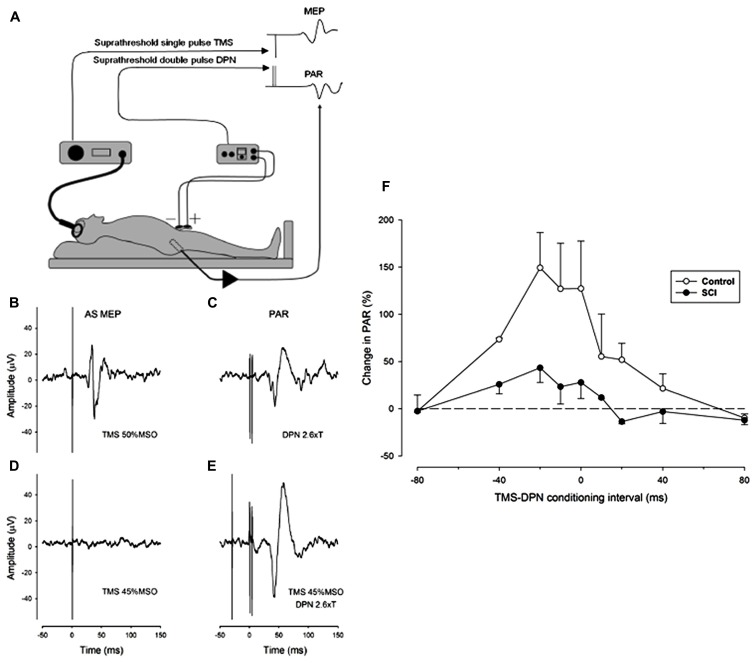
**Facilitation of the pudendal anal reflex (PAR) by single pulse transcranial agnetic stimulation (TMS) of the motor cortex. (A)** Experimental set-up showing position of dorsal penile nerve stimulating electrodes (DPN), anal sphincter EMG recording electrode and the TMS double cone coil. Insets: idealized PAR and anal sphincter motor evoked potential (MEP) responses. **(B-E)** Anal sphincter EMG averaged (*n* = 10) evoked responses in a control subject. **(B)** MEP to cortical TMS at 50% maximum stimulator output (MSO). **(C)** PAR response to stimulation of the DPN at 2.6 times sensory threshold (18 mA). **(D)** Lack of response to TMS at 45% MSO. **(E)** TMS at 45% MSO preceding DPN at 2.6 times sensory threshold by 30 ms. The DPN stimulus in E now elicits an enhanced PAR that is approximately double the peak-to-peak size of the unconditioned PAR in **(C)**. **(F)** The degree of facilitation of the PAR by TMS at different conditioning intervals. Negative intervals indicate that the TMS occurred prior to the DPN stimuli. The graph presents the average (mean ± SE) percentage increase in the PAR for three control (open symbols) and three iSCI (closed symbols) subjects. The dashed horizontal line represents a level of zero facilitation. The degree of facilitation is significantly greater for the control group (Wilcoxon signed rank test *P* = 0.008).

We have adopted a practical proposal for the study of the interaction between reflex and voluntary control of sphincter musculature which is to substitute the PAR as a surrogate marker for the urethral sphincter guarding reflex (Podnar and Vodusek, 2001). The approach has been coupled with the use of single pulse TMS of the motor cortex to stimulate the corticospinal system and elicit a MEP in the external anal sphincter. To explore the natural interaction between reflex and voluntary control of the sphincter muscle we have conditioned the PAR by prior single pulse TMS of the motor cortical representation of the anal sphincter muscle in both neurologically normal (control) subjects and in iSCI subjects with a neurogenic bladder. Preliminary findings in three control and three iSCI subjects showed facilitation of the PAR by TMS at strength close to threshold for eliciting an MEP (Craggs et al., 2007). In both groups facilitation was observed with a wide range of conditioning intervals, the maximum amount of facilitation occurring when TMS preceded stimulation of the dorsal penile nerve (DPN) by intervals between 20 and 40 ms (see **Figure 1**). Some facilitation is evident even when DPN stimulation slightly precedes TMS. This does not preclude a corticospinal-motoneuronal site of interaction as the latency of the PAR is invariably longer than any TMS elicited MEP by 5-15 ms. The amount of facilitation was greater in the control subjects.

A study was then carried out on a further cohort of 23 iSCI subjects with a neurogenic bladder to see the extent to which conditioning the PAR by TMS with a fixed interval of 30 ms would elicit facilitation (Vasquez et al., 2014). Only 12 of the subjects showed facilitation of the PAR to TMS applied 30 ms before DPN stimulation. An anal sphincter MEP could be elicited in 8 of those 12 in response to TMS alone using strength of TMS up to the maximum that could be tolerated by the subjects. MEPs could similarly be elicited in a further five subjects who failed to show facilitation of the PAR. These results high-light some of the confounding issues raised earlier with regard to the use of TMS in SCI. It was not possible in this study to know whether the absence of facilitation or an MEP was due to interruption of the relevant neural pathways or whether the strength of TMS was inadequate. Whatever the reason for the presence or absence of demonstrable facilitation, the results suggest that it could be appropriate to screen iSCI subjects for cortical facilitation of the PAR before undertaking any plasticity inducing regime designed to strength the corticospinal connections involved.

Having determined the characteristics of corticospinal facilitation of the PAR, and the frequency with which it can be elicited in iSCI, the intention is to see whether either high frequency (5 Hz) rTMS or PAS (paired DPN and TMS) might reverse any maladaptive reflex plasticity induced by SCI and elicit persistent changes in either the PAR, the anal sphincter MEP or facilitation of the PAR conditioned by single pulse TMS. The preliminary study will be carried out in subjects with iSCI on the premise that the residual corticospinal connections, possibly already having been subjected to plastic change as a result of the injury (de Groat and Yoshimura, 2012), might be more responsive than unaffected circuits in control subjects. Initially, there is the consideration as to whether an rTMS protocol will prove practical for use with iSCI subjects. The following problems to be expected with application of rTMS are (1) the high motor threshold found in most iSCI subjects that results in unacceptable levels of stimulation, (2) the motor threshold of some iSCI subjects being beyond maximum output of the magnetic stimulator, and (3) high magnetic stimulator outputs causing over-heating of magnetic coils resulting in durations of rTMS deemed too short to be effective. These were confounding issues for application of rTMS in iSCI described previously in this article. Alternatively, a paired associative stimulation (PAS; Stefan et al., 2000) protocol might be a more practical plasticity-inducing for iSCI in that it would not require high frequencies of TMS. Such an application of PAS targeting cortical plasticity has yet to be applied in iSCI although Bunday and Perez (2012) have successfully employed spike-timing dependent stimulation to induce plasticity in cortico-motoneuronal synapses at the level of the spinal cord (see above). In order to gage the appropriate PAS interval between peripheral nerve and cortical stimulation that could induce cortical plasticity in the circuits controlling sphincter function, the conduction time of pudendal afferents to somatosensory cortex has to be determined. The earliest identifiable component of the cortical somatosensory evoked potential (SSEP) to electrical stimulation of the DPN is a positive peak (P1) with a latency ranging from 39 to 46 ms in the normal population (Kaiser et al., 2001). The pudendal SSEP has a form and latency very similar to that evoked by stimulation of the posterior tibial nerve at the ankle. However, the pathway from the pudendal nerve is shorter and it has been estimated that the spinal conduction time is correspondingly longer than for tibial nerve stimulation (Choi et al., 2001). There appears to be no obvious earlier N1 component to the pudendal nerve SSEP, which is thought to represent arrival at the somatosensory cortex of an afferent volley elicited by upper limb nerve stimulation (Allison et al., 1989) and which leads to activation in the primary motor cortex approximately 4 ms later (Goldring et al., 1970). However, it has been possible to reason from the measure of spinal and intracranial conduction times that the P1 component of the pudendal SSEP represents cortical activation (Guerit and Opsomer, 1991; Choi et al., 2001). On that basis it is reasonable to expect that an appropriate PAS interval (to induce plasticity at a cortical level) between DPN stimulation and TMS directed at the cortical representation of the anal sphincter should be around 40 ms. However, this would not take into account the fact that afferent spinal conduction time may well be delayed by SCI. We anticipate that the most appropriate PAS interval would need to be tailored to individual iSCI subjects according to whether the P1 component of the pudendal nerve (DPN) SSEP is delayed.

Finally, other factors such as repetition rates of paired stimuli and time of day for administration of PAS may require consideration in order to maximize plasticity and reduce variability (Sale et al., 2007).

## CONCLUSION

The application of repetitive forms of TMS to obtain functional benefits by inducing favorable plastic changes in residual corticospinal circuits following iSCI has received little attention compared to other neurological conditions such as stroke, depression and neuropathic pain (see Ridding and Rothwell, 2007). This may in part be attributed to confounding issues to the use of rTMS relating to the relatively high motor thresholds to TMS in iSCI that are the consequence of damage to the corticospinal tract. Individual variation in the extent and level of SCI and the consequent variability in impact on sensorimotor control also create difficulty in formulating plasticity inducing TMS protocols. It is likely that emerging strategies will need to have more rigorous inclusion selection criteria based on clinical and neurophysiological sensorimotor assessment of iSCI subjects, particularly for novel approaches such as restoration of sphincter function, and tailor the rTMS protocol to the properties of the residual corticospinal circuits of individuals.

## Conflict of Interest Statement

The authors declare that the research was conducted in the absence of any commercial or financial relationships that could be construed as a potential conflict of interest.

## ABBREVIATIONS

AIS: American spinal injuries association (ASIA) impairment scale
ARAT: action research arm test
EMG: electromyography
DPN: dorsal penile nerve
DSD: detrusor sphincter dyssynergia
EPT: electrical perceptual threshold
iSCI: incomplete spinal cord injury
ISNCSCI: international standards for neurological classification of spinal cord injury
LT: light touch
MAS: modified Ashworth scale
MEP: motor evoked potential
NDO: neurogenic detrusor over-activity
PAR: pudendo-anal reflex
PP: pin prick
SCI: spinal cord injury
SSEP: somatosensory evoked potential
TMS: transcranial magnetic stimulation
rTMS: repetitive transcranial magnetic stimulation
TUG: timed up and go test
U&LEMS: upper and lower extremity motor scores
WISCI II: walking index for spinal cord injury, version II
% MSO: percentage of maximum stimulator output.
